# Comparison of methods for estimation of genetic covariance matrix from SNP or pedigree data utilised to predict breeding value

**DOI:** 10.1186/1753-6561-4-s1-s7

**Published:** 2010-03-31

**Authors:** Sebastian Mucha, Anna Wolc, Tomasz Strabel

**Affiliations:** 1 Department of Genetics and Animal Breeding, Poznan University of Life Sciences, Wolynska 33, 60-637 Poznan, Poland

## Abstract

**Background:**

The aim was to predict breeding values of non-phenotyped individuals based on a dataset prepared for the 13^th^ QTL-MAS Workshop in Wageningen.

**Methods:**

Genetic co-variance matrices between animals were estimated with three methods: one using pedigree information only and two based on SNP markers from the first chromosome. Quadratic regression of breeding values, estimated separately in each of the five time points, was used to predict the breeding values in the 6^th^ time point.

**Results:**

Based on the comparison (true - estimated BV) it can be concluded that SNP based methods provided better estimates (accuracy between 0.75 and 0.80) than pedigree (0.65).

**Conclusions:**

Even though only SNPs from chromosome 1 were used it was still possible to achieve fairly high accuracies. Most likely this was due to the fact that chromosome 1 contained the QTLs with the largest effects.

## Background

The analysis was based on a dataset prepared for the 13^th^ QTL-MAS Workshop in Wageningen [1]. The aim of this paper was to predict breeding values of the 1000 non-phenotyped animals in the 6^th^ time point, using three different strategies based on similarity between individuals due to common ancestry (pedigree records), and two methods based on marker similarity. Due to software limitations [2] only one chromosome could be included in the analysis. The first chromosome was chosen based on preliminary results of QTL mapping, performed with a single QTL model with additive effects in the GRID QTL package [3]. The most significant QTLs, affecting the analysed trait in all five time points, were found on chromosome 1.

## Methods

### Estimation of genetic relationship

Genetic covariance matrices between all animals present in the dataset were estimated with three methods. First approach (pedigree based method - PB) was based on the additive relationship matrix calculated from pedigree. Second method computed similarity between individuals as a correlation coefficient between allelic states using 90 SNP markers from chromosome 1 (SNP based method - SNPL). For this purpose the method of Loiselle [4] was used as implemented in software package SPAGeDi 1.2g [2], which computes relationship as a_ij_ = Σ1	[ Σa(Σc_i_Σc_j_(*x*_1cia_ - *p*_1a_)(*x*_1cja_ - *p*_1a_)/Σc_i_Σc_j_1) + Σa(*p*_1a_(1 - *p*_1a_)/(*n*_1_ - 1)) ] / ΣlΣa (*p*_1a_(1 - *p*_1a_)) where x_1cia_ is an indicator variable (x_1cia_ = 1 if the allele on chromosome* c* at locus* l* for individual* i* is *a*, otherwise x_1cia_ = 0), p_1a_ is the frequency of allele* a* at locus* l* in the reference sample,* n*_1_ is the number of alleles defined in the sample at locus* l* (the number of individuals times the ploidy level minus the number of missing alleles), and Σc_i_ stands for the sum over the homologous chromosomes of individual *i*. Here, the term involving (*n*_1_ - 1) is a sampling bias correction. The program calculates the pair wise relationship between animals i and j (a_ij_) leaving the diagonal elements blank (a_ii_), thus selfcoancestry had to be estimated as: F_k_ = 1 + 0.5*a_ij_, where a_ij_ is relationship between parents i and j of individual k.

The third method used MCMC (Markov Chain Monte Carlo simulations) to estimate genetic relationship between animals for a selected number of 39 SNP markers from chromosome 1, with minor allele frequency above 0.1 (selected SNP method - SNPC). This limitation was imposed due to time-extensive properties of the MCMC method. Software package Citius[5,6] was used to apply the MCMC method to calculate multilocus genotype probabilities and to analyse genes shared identical by descent (IBD). IBD matrices were calculated in 9 points along the analysed fragment of chromosome 1. Afterwards they were averaged into one G-matrix, that was used for further computations.

### Estimation of variance components and breeding values

Variance components were estimated separately for each time point (0, 132, 265, 397, 530), with ASREML [7] using the following model:

*y_i_* = *μ* + *a_i_* + *e_i_*

Where: y_i_ - analysed trait a_i_ - random additive genetic effect of animal i; e_i_ - random residual effect.

The covariance structure was specified as:

* and  *

where: - additive genetic variance,  - residual variance, G - genetic relationship matrix, I – identity matrix.

The analysis was performed with three types of genetic covariance matrices (G) based on: pedigree (PB method), and SNP markers (methods SNPL and SNPC).

Quadratic regression of predicted breeding values on time, extracted from ASREML, in the first five time points was applied to estimate least square regression coefficients for each animal. Subsequently, the estimated regression coefficients were used topredict the unknown breeding values in the 6^th^ time point (time 600) using the following formula:

where: y_600,i_ is the breeding value in time point 600; , , *and,* are least square regression coefficients, estimated for animal i.

## Results

### Variance components

Regardless of the method used, genetic and residual variances increased with time (Table 1). Estimates of genetic variance were lower for the SNPC method than the PB or SNPL method. In case of the PB method heritability decreased from 0.51 (time point 0) to 0.47 (time point 530). On the other hand the SNPL method resulted in genetic variance increasing more than the residual variance, which resulted in an increase of heritability from 0.53 (time point 0) to 0.60 (time point 530). Heritability estimates for the SNPC method did not differ much between the time points and were between 0.40 and 0.41. Correlations between breeding values in the different time points were high - between 0.82 and 0.99 for the SNPL and SNPC methods (Table 2 and Table 3) and between 0.79 and 0.99 for the PB method (Table 4) reached around 0.98.

**Table 1:** Estimates of genetic and residual variance and heritability at five time points (T0, T132, T265, T397 and T530) obtained with three methods: SNPL - covariance structure from 90 SNPs on chromosome 1 (Loiselle et al. 1995), SNPC - covariance structure from 30 selected SNPs with minor allele frequency >0.1 (Szydlowski et al. 2008), PB - covariance structure from pedigree.

	T0	T132	T265	T397	T530
**Genetic variance**
PB	0.05	0.33	1.81	7.49	19.23
SNPL	0.05	0.35	2.10	8.84	23.32
SNPC	0.04	0.28	1.62	6.50	16.21
**Residual variance**
PB	0.05	0.35	2.05	8.62	21.32
SNPL	0.04	0.27	1.53	6.35	14.98
SNPC	0.06	0.41	2.30	9.58	23.76
**Heritability**
PB	0.50	0.48	0.46	0.46	0.47
SNPL	0.53	0.56	0.57	0.58	0.60
SNPC	0.40	0.40	0.41	0.40	0.40

**Table 2:** Correlation between breeding values in five time points (T0, T132, T265, T397 and T530) , estimated with a genetic covariance matrix based on 90 SNPs from chromosome 1 (SNPL method).

	T0	T132	T265	T397	T530
T0					
T132	0.987				
T265	0.939	0.981			
T397	0.862	0.929	0.982		
T530	0.818	0.894	0.961	0.995	

**Table 3:** Correlation between breeding values in five time points (T0, T132, T265, T397 and T530) estimated with a genetic covariance matrix based on a selected number of SNPs from chromosome 1, with minor allele frequency >0.1 (SNPC method).

	T0	T132	T265	T397	T530
T0					
T132	0.987				
T265	0.940	0.981			
T397	0.865	0.932	0.983		
T530	0.824	0.899	0.964	0.995	

**Table 4:** Correlation between breeding values in five time points (T0, T132, T265, T397 and T530) estimated with a genetic covariance matrix based on pedigree records (PB method).

	T0	T132	T265	T397	T530
T0					
T132	0.986				
T265	0.930	0.978			
T397	0.841	0.918	0.979		
T530	0.792	0.879	0.956	0.994	

**Table 5:** Correlation and regression of true breeding values (provided by the organizers) on breeding values estimated with three methods: PB - relationship based on pedigree records, SNPL - genetic similarity estimated from 90 SNP markers from chromosome 1, SNPC - relationship estimated from selected SNP markers from chromosome 1 with minor allele frequency >0.1.

	PB	SNPL	SNPC
Correlation	0.65	0.75	0.80
Regression	0.93	0.79	0.92

### Breeding values

**Figure 1:**
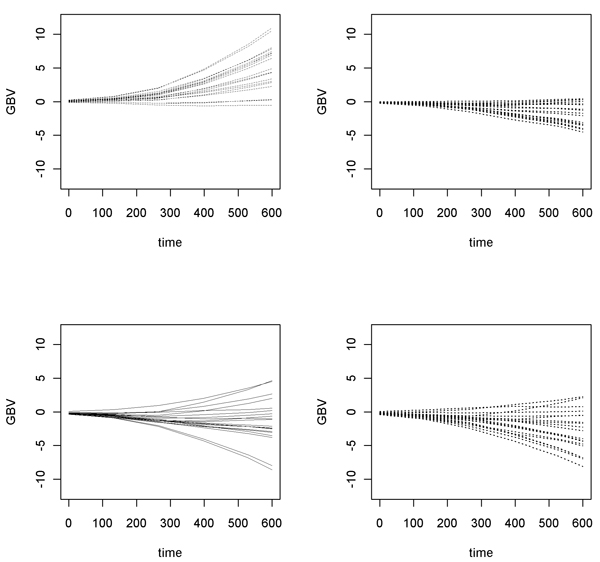
Breeding values for members of the same 4 arbitrary selected full sib families (top diagrams - families without observations; bottom diagrams - families with observations) estimated with genetic covariances based on 90 SNP markers from chromosome 1 (SNPL method). Similar picture was obtained when only 30 SNPs, with minor allele frequency >0.1, from chromosome 1 (SNPC method) was used (result not shown)

The variance of predicted breeding values in the time point 600 was the lowest for the PB method (17.44), the highest for the SNPL method (27.94), and moderate for the SNPC method (20.42). In contrast to the PB in case of the SNP based methods there was considerable variation in breeding values within FS families (Figure 1). Correlations between breeding values in time point 600, estimated with the three methods were between 0.82 and 0.86. When comparing the list of top 20 nonphenotyped animals selected with the three methods with the true list of top 20 nonphenotyped animals from the simulation (as provided by organizers), than the PB method had only 30% of individuals in common. Higher agreement was found for SNP based methods: 45% and 50% for the SNPC and the SNPL methods, respectively. Accuracy of breeding values (correlation between predicted and true breeding values) for the 1000 nonphenotyped individuals in the time point 600 was the lowest for the PB method (0.65), higher for the SNPL method (0.75) and the highest for the SNPC method (0.80). Regression coefficients of true breeding values on predicted breeding values were between 0.79 - 0.93 (Table 5).

## Discussion

The genetic variance estimated with the PB method was the closest to the true (simulated) one while variance components obtained with SNPL method were slightly overestimated. Underestimation of genetic variance with the SNPC method can be due to ignoring a part of SNPs. Changes of heritability estimates in case of the SNPL method could be due to overestimation of genetic variance which was higher for higher phenotypic variance. Genomic breeding values usually show bias, which is a consequence of using marker instead of QTL effects [8]. This bias exist also in our results - regression of true breeding values on predicted breeding values is much below 1.

Rather high variation of breeding values could be partly due to Mendelian variation and partly as a result of method inadequacy. Both SNPL and SNPC methods explored differences among animals within full-sib families but it is hard to decide which one should be preferred as one yielded higher accuracy but the second more correctly chose the top 20 animals.

It is also worth mentioning that the method for prediction of breeding values applied in this paper (quadratic regression) does not take into account the fact that the analysed trait will eventually reach its asymptotic value.

The restriction of using only one chromosome was imposed partially due to the fact that SNPC method is very computationally demanding and the SNPL method had a software limitation for the number of markers. This might have a drawback of our analysis as it neglects large part of the available SNP information. Nevertheless this simplified analysis allowed to predict breeding values with fairly high accuracy of 0.75 - 0.80. However because after the analysis it turned out that the first chromosome contained QTLs with the largest effect [1] it may be concluded that the result would have been much worse in other, practical situations.

In our analysis we used a concept of genetic relationship matrix to obtain genomic breeding values, which is similar to the method described by Zhang et al. [9]. Van Raden showed that reliabilities of GEBVs based on this approach are almost as high as in the Bayes B method [10]. The SNPL and SNPC methods, both assumed equal effects of all markers from chromosome I.

## Conclusions

Application of SNP markers enables to differentiate breeding values within full sib families. Based on the comparison of true (simulated by organizers) breeding values in the time point 600 with our predictions it can be concluded that SNP based methods provided relatively good estimates. Even though only SNPs from chromosome 1 were used it was still possible to achieve fairly high accuracies. Most likely it was due to the fact that chromosome 1 contained the most significant QTLs affecting the analysed trait.

## Competing interests

The authors declare that they have no competing interests.

## Authors' contributions

SM: performed the analysis and drafted the manuscript. AW: performed the analysis and drafted the manuscript. TS: drafted the manuscript. All authors read and approved the final manuscript.
